# Epistaxis as a complication of high-flow nasal cannula therapy in adults

**DOI:** 10.5935/0103-507X.20210090

**Published:** 2021

**Authors:** Viviane Cordeiro Veiga, Lígia Maria Coscrato Junqueira Silva, Érica Regina Ribeiro Sady, Israel Silva Maia, Alexandre Biasi Cavalcanti

**Affiliations:** 1 Intensive Care Unit, BP-A Beneficência Portuguesa de São Paulo - São Paulo (SP), Brazil.; 2 Intensive Care Unit, Hospital Nereu Ramos - Florianópolis (SC), Brazil.; 3 Research Institute, HCor-Hospital do Coração - São Paulo (SP), Brazil.

**To the Editor**

## INTRODUCTION

High-flow nasal cannula (HFNC) therapy has emerged as a valuable therapy for adult patients with acute respiratory failure^(^1^,^2^)^ or to prevent postextubation respiratory failure.^(^3^,^4^)^ Currently, HFNC therapy is recommended in guidelines on the management of patients with coronavirus 2019 disease (COVID-19).^(^5^)^ HFNC therapy delivers humidified and heated gas at a high flow that can exceed the patient’s inspiratory demand of flow, contributing to alleviating breathlessness.^(^6^)^ It promotes washout of the nasopharyngeal space, creating a pharyngeal reserve of fresh gas for subsequent inspiration.^(^7^)^ These mechanisms result in improved oxygenation and reduced work of breathing.^(^8^)^ Clinical complications of HFNC therapy have rarely been reported.^(^9^)^ Some studies have described mild complaints, such as feeling too warm, unpleasant smell or thoracic discomfort.^(^9^)^ Epistaxis is a rare adverse event associated with HFNC therapy in children.^(^10^)^ However, HFNC-related epistaxis in adults has been reported only in one patient under higher than recommended flow (65L/min).^(^11^)^ Here, we report 7 cases of epistaxis we observed in a series of 70 adults treated with HFNC therapy.

## METHODS

Retrospective case series including all adult patients treated with HFNC therapy in the intensive care unit (ICU) over a 1-year period (September 2017 to October 2018). Indications for HFNC therapy were support to patients with acute respiratory failure or to prevent postextubation failure. The study was approved by the Research Ethics Committee of *BP-A Beneficência Portuguesa de São Paulo*. High-flow nasal cannula therapy was delivered via Precision Flow^®^ (Vapotherm, Inc, Exeter, NH) using small-bore nasal cannulas, sizes 2.7mm and 4.8mm. Flow was initiated at 30L/minute with adjustments up to 40L/minute aiming to reduce respiratory distress. The fraction of inspired oxygen (FiO_2_) was adjusted to maintain peripheral oxygen saturation higher than 92%. The temperature was adjusted between 35ºC and 37º Data were extracted from the electronic medical records of patients. We collected data on the following variables at ICU admission: age, sex, main diagnosis, comorbidities, severity of illness (Simplified Acute Physiology Score 3 - SAPS 3), Sequential Organ Failure Assessment (SOFA) score, and type of acute respiratory failure (hypoxemic or hypercapnic). We obtained the following data at the initiation of HFNC therapy: respiratory characteristics (the ratio of peripheral oxygen saturation - SpO_2_ to FiO_2_, respiratory rate, and the ROX index - a ratio of SpO_2_/FiO_2_ to respiratory ratio), HFNC therapy settings (FIO_2_, flow and temperature), and the results of selected laboratory tests (activated partial thromboplastin time - aPTT, prothrombin time - PT, and platelets). We collected data on the following clinical outcomes during the ICU stay: epistaxis, failure of HFNC, mortality and length of stay.

## RESULTS

A total of 70 patients were treated with HFNC therapy in this period (Table 1). Seven patients (10%) developed epistaxis while on HFNC therapy. Median age was 67 years and 40% of patients were female. A HFNC was indicated for support in acute respiratory failure in 84% of cases. The distribution of diagnosis was apparently different between the patients with and without epistaxis (p = 0.02), with a lower prevalence of pneumonia and a higher prevalence of nonpulmonary sepsis in patients with epistaxis. The mean SAPS 3 score was 53.9 (standard deviation - SD 16.8), the mean SOFA score was 9.2 (SD 1.7), and 14 patients (20%) were on vasopressors, without statistically significant differences for these variables. There were no statistically significant differences between patients with and without epistaxis in platelet count and aPTT, Although International Normalized Ratio (INR) was slightly lower among patients with epistaxis (1.2 *versus* 1.3; p = 0.03). There were no statistically significant differences between patients with and without epistaxis in HFNC settings (flow, temperature and FiO_2_) at initiation and at end of therapy (Table 2). There also were no differences in the duration of HFNC therapy. There were no statistically significant differences in the incidence of adverse clinical outcomes (need for mechanical ventilation, ICU and hospital mortality, as well as ICU and hospital length of stay) between patients with and without epistaxis (Table 3). Significant epistaxis occurred in only one patient, and it requiring an epistaxis device (Rapid Rhino^©^). Two other cases used topical epinephrine. There was no need for transfusion of blood products in any of the cases.

**Table 1:** Characteristics at intensive care unit admission of all patients receiving high-flow nasal cannula therapy and those with and without epistaxis

Variable	Total n = 70	Epistaxis n = 7	No epistaxis n = 63	p value
Age (years)	67 [58.2 - 80]	80 [58 - 84]	66 [58.5 - 78]	0.445
Female	28 (40)	4 (57.1)	24 (38.1)	0.426
Indication for HFNC				0.587
Acute respiratory failure	59 (84.3)	7 (100)	52 (82.5)	
Prevention of postextubation failure	11 (15.7)	0 (0)	11 (17.5)	
Diagnosis				0.02
Pneumonia	32 (45.7)	0 (0)	32 (50.8)	
Nonpulmonary sepsis	11 (15.7)	3 (42.9)	8 (12.7)	
Exacerbated COPD	3 (4.3)	1 (14.3)	2 (3.2)	
Cardiogenic pulmonary edema	7 (10)	1 (14.3)	6 (9.5)	
Pulmonary embolism	4 (5.7)	1 (14.3)	3 (4.8)	
Abdominal postoperative	3 (4.3)	0 (0)	3 (4.8)	
Cardiovascular postoperative	10 (14.3)	1 (14.3)	9 (14.3)	
Comorbidities				
COPD	7 (10)	0 (0)	7 (11.1)	1
Stroke	10 (14.3)	1 (14.3)	9 (14.3)	1
Chronic renal failure	14 (20)	5 (71.4)	9 (14.3)	0.003
Hepatic disease	4 (5.7)	1 (14.3)	3 (4.8)	0.35
Heart failure	7 (10)	2 (28.6)	5 (7.9)	0.142
Hematological malignancies	9 (12.9)	0 (0)	9 (14.3)	0.583
Solid tumor	23 (32.9)	3 (42.9)	20 (31.7)	0.676
Use of vasopressors	14 (20)	3 (42.9)	11 (17.5)	0.137
SAPS3*	53.9 ± 16.8	61 ± 17	53.2 ± 16.7	0.244
SOFA†	9.2 ± 1.7	9.1 ± 1.1	9.2 ± 1.8	0.945
Platelets	142 [81.8 - 222.5]	133 [47 - 143.5]	148 [84.5 - 226]	0.313
INR	1.3 [1.2 - 1.4]	1.2 [1.1 - 1.2]	1.3 [1.2 - 1.4]	0.026
aPTT	32 [28.5 - 38.5]	34 [30.9 - 40]	31.8 [28.4 - 38]	0.776

HFNC - high-flow nasal cannula; COPD - chronic obstructive pulmonary disease; SAPS - Simplified Acute Physiology Score; SOFA - Sequential Organ Failure Assessment; INR - International Normalized Ratio; aPTT - activated prothrombin time.

* Range, 0 to 217; higher scores indicate higher severity of illness and risk of in-hospital death. † Range, 0 to 24; higher scores indicate a greater severity of organ dysfunction in critically ill patients and risk of in-hospital death (e.g., a score of 10 predicts an in-hospital mortality of 50%). Results expressed as median [interquartile range], n (%), or mean ± standard deviation.

## DISCUSSION

We observed epistaxis as an adverse event occurring in 7 of 70 patients administered HFNC therapy. Reasons for using HFNC therapy were diverse, and none of the baseline characteristics of patients were associated with epistaxis. Initial and final HFNC settings were also not associated with epistaxis. However, the number of epistaxis events was small; therefore, our study has limited power to identify risk factors. In addition to this study, only one study by Velasco Sanz et al.^(^11^)^ reported epistaxis in one adult from a series of 12 administered HFNC therapy. The authors attributed the adverse event to the high flow rate in use (65L/minute). In our study, the maximum flow rate was 40L/minute and was not different between patients with or without epistaxis. Baudin et al.^(^10^)^ reported complications associated with the use of HFNC therapy in a retrospective observational study in critically ill children. Significant epistaxis occurred in only one patient, without identifying potential causes for the adverse event.

Small-bore nasal cannulas promote faster purging of the extrathoracic dead space with lower flow rates than large-bore nasal cannulas.^(^12^)^ This happens because of a smaller prong configuration at the tip that increases the velocity of the gas. A possible explanation for epistaxis is the jetting effect from the tip of the cannula, which could result in undue shear stress on the mucosal tissue of the airway.

There are several limitations in this study. First, we reported a small case series with only 7 patients experiencing the outcome. Therefore, the precision around our incidence estimate is large. In addition, the study has limited power to identify risk factors for bleeding. Second, HFNC therapy was assessed in many clinical trials and case series involving hundreds of patients, with only 1 previous case of epistaxis being reported. Therefore, the true incidence of epistaxis among patients administered HFNC therapy is likely lower than the number (10%) we observed in our study.

**Table 2:** High-flow nasal cannula initial and final settings of all treated patients those with and without epistaxis

Variable	Total n = 70	Epistaxis n = 7	No epistaxis n = 63	p value
Flow (L/minute)				
Initial	30 [24 - 40]	30 [22.5 - 30]	30 [24 - 40]	0.49
Final	25 [20 - 38.8]	20 [19 - 29]	25 [20 - 40]	0.23
Temperature (°C)				
Initial	36 [34.2 - 36]	36 [35.5 - 36]	35 [34 - 36]	0.20
Final	36 [34 - 36]	36 [34 - 36]	36 [34 - 36]	0.90
Fraction of inspired oxygen				
Initial	0.50 [0.40 - 0.75]	0.40 [0.40 - 0.55]	0.55 [0.40 - 0.75]	0.34
Final	0.40 [0.30 - 0.70]	0.50 [0.35 - 0.60]	0.40 [0.30 - 0.70]	0.98
Duration of HFNC therapy (days)	2.5 [1 - 5]	4 [0.5 - 11.5]	2 [1 - 5]	0.59

HFNC - high-flow nasal cannula. Results expressed as median [interquartile range].

**Table 3:** Clinical outcomes of all patients receiving high-flow nasal cannula therapy those with and without epistaxis

Variable	Total n = 70	Epistaxis n = 7	No epistaxis n = 63	p value
Need of mechanical ventilation after HFNC therapy	33 (47.1)	3 (42.9)	30 (47.6)	1
ICU mortality	30 (42.9)	3 (42.9)	27 (42.9)	1
ICU length-of-stay (days)	12 [7 - 22]	21 [6 - 22]	12 [7 - 20.5]	0.92
In-hospital mortality	40 (57.1)	4 (57.1)	36 (57.1)	1
In-hospital length-of-stay (days)	28.5 [16 - 48.5]	28 [15 - 33]	29 [16.5 - 49.5]	0.41

HFNC - high-flow nasal cannula; ICU - intensive care unit. Results expressed as n (%) or median [interquartile range].

## CONCLUSION

In conclusion, epistaxis is a possible complication related to the use of a high-flow nasal cannula. In our small study sample, none of the characteristics of patients or high -flow nasal cannula settings were associated with epistaxis.
